# Preparation of Pure Fatty Acids for the Manufacture of Candles by the Distillation of Common Fats

**Published:** 1852-10

**Authors:** 


*Preparation of pure fatty acids for the manufacture of candles by the distillation of common fats.*—At the last Conversazione, held at the house of the Pharmaceutical Society, a series of specimens were exhibited illustrating the process adopted at the works of Price's Patent Candle Company for obtaining a white and hard fatty substance, suitable for the manufacture of the best description of candles by distillation from palm oil and other cheap fats. The following description of the details of the process is given in the "Reports of the Juries" of the Great Exhibition.

*Sulphuric Saponification.*—About twenty tons of fat, say palm oil, are placed in a large lead-lined vat, and fused by a steam jet. The fluid mass, after being allowed to settle, has now to be exposed to the combined action of concentrated sulphuric acid and heat, and for this purpose is pumped up into the acidifying vessel, in which its temperature is raised to 177° Cent. (350° Fah.) The means of heating is a jet of low-pressure steam, which, in its course from the boiler, passes through a series of iron pipes heated in a furnace. The quantity of acid used is in the proportion of 6 lbs. for 112 lbs. of palm oil. In this operation the palm-oil is decomposed and becomes much blackened. Withdrawn at that period it is seen that an important change has been affected by the action of the acid, as the mass now really crystallizes to a tolerably solid fat. The fat is now drawn off from the acid and transferred to the washing-tank, where it is boiled up with water by means of a steam-jet.

*Distillation.*—After one or two washings the blackened fat is withdrawn, and pumped up to the supply tank, which commands the stills. The stills which are made of copper, are heated by an open grate; each still is capable of holding five tons of fat. When charged the temperature is raised to 293.5° Cent. (560° F.), and low pressure steam passed through the mass. This steam is previously heated by passing through a system of iron pipes placed in a furnace.

The current of steam carries with it the vapor of the fatty acids, and thus facilitates the process. The mixed vapors of fatty acids and water pass together to a series of vertical pipes, which retain a temperature above 100° Cent. 212° F.) where the fats only condense while the steam passes to a second refrigerator, cooled by a current of water; here it is condensed along with the minute quantity of fat carried over by it. A separating tank, from which the water escapes at the bottom, whilst the fats float on the top, serves to recover this small quantity.

*Distillation of the Residue.*—After continuing the distillation for a certain period, the residue in the still is transferred to another still, formed of iron pipes, set in a furnace, and there submitted to a much higher temperature, and a jet of steam more strongly heated. The residue left in these iron stills is a sort of pitch, and is applied to the same uses as ordinary pitch. By this means an additional quantity of fatty acids is obtained.

The fatty acids, as they run from the still, are used to a great extent for the manufacture of candles, without pressing, and form what are called *composite candles*, which possess all the advantages of being self-snuffing, but are more fusible and softer than the pressed stearic acid candles.

A large proportion of the distilled fats, however, is pressed, to make a better sort of candle, and for this purpose fifty hydraulic presses are employed.

*Cold Pressing.*—The fats are spread by ingenious machinery on woven mats, and submitted to powerful cold-pressure between iron plates; the oleic, or metoleic acid runs out, and is collected, and chiefly exported to Germany, where it is employed in soap-making.

*Hot-Pressing.*—After cold-pressing, the fat acids are subjected to hot pressure in hydraulic presses, confined in a chamber heated by steam. The pressed cakes, after the removal of the edges are melted in contact with a little diluted sulphuric acid, and run into blocks. When the reporters visited the works the Company were distilling at the rate of 130 tons of palm oil per week.—*Ibid.*

